# Association between the modified cardiometabolic index and asthma among participants in the United States: A cross-sectional NHANES analysis

**DOI:** 10.1097/MD.0000000000047444

**Published:** 2026-01-30

**Authors:** Yuan Fang, Man Wang, Yuehu Liu, Zi Lin, Xiaoqin Wang, Qin Zhang

**Affiliations:** a Department of Neonatology II, Wuhan Children’s Hospital, Wuhan Maternal and Child Health Hospital, Tongji Medical College, Huazhong University of Science and Technology, Wuhan, Hubei, China; b School of Nursing, Wuhan University, Wuhan, China.

**Keywords:** asthma, cardiometabolic health, cross-sectional NHANES analysis, modified cardiometabolic index, US participants

## Abstract

The modified cardiometabolic index (MCMI) integrates key cardiometabolic factors that drive airway inflammation in asthma through abnormal adipokine secretion, lipid disorders, and impaired glucose metabolism. This study aimed to explore the association between the MCMI and the odds of self-reported asthma in the United States. This cross-sectional analysis utilized data from the National Health and Nutrition Examination Survey, which includes demographic, laboratory, health, and comorbidity information. Participants who self-reported asthma and had MCMI scores were included in the study. We employed multivariate logistic regression and subgroup analyses to examine the relationship between MCMI and the odds of self-reported asthma. Four models were adjusted to account for potential confounders, providing deeper insights into the independent associations between MCMI and the odds of self-reported asthma. Sensitivity analyses were performed to validate the robustness of these associations. Among 16,111 participants (mean age: 50.1 ± 17.9 years), 2137 (13.3%) reported asthma. Univariate analysis showed a positive correlation between MCMI and the odds of self-reported asthma (OR = 1.17, 95% CI: 1.11–1.24, *P* < .001). This relationship remained significant after adjusting for several variables. Compared to the Q1 MCMI group, adjusted odds ratios for Q2, Q3, and Q4 were 0.95 (95% CI: 0.83–1.08, *P* = .439), 0.99 (95% CI: 0.87–1.13, *P* = .920), and 1.30 (95% CI: 1.14–1.47, *P* < .001) in model 1, respectively. Moreover, curve fitting analysis revealed a significant linear correlation between MCMI and the odds of self-reported asthma. The results of the sensitivity, subgroup, and stratified analyses were robust, confirming the stability and reliability of the observed associations. A modest positive association exists between MCMI and the odds of self-reported asthma in the United States. Further research is required to explore the underlying mechanisms and potential clinical applications.

## 1. Introduction

Asthma is a chronic condition characterized by episodic wheezing, coughing, and breathlessness stemming from airway hyperresponsiveness and inflammation. It affects approximately 300 million people globally, and its prevalence is continuously increasing.^[1]^ Asthma severely impacts the quality of life of those affected and imposes a substantial burden on society and healthcare systems. In severe cases, it can be fatal. Although the etiology of asthma is multifactorial, involving genetic, environmental, and lifestyle factors, metabolic dysregulation plays a pivotal role in asthma pathogenesis.^[2,3]^ Abnormal adipokine secretion due to adiposity drives airway inflammation. Dysregulated lipids, via pro-inflammatory mediator release, further amplify this inflammatory state. Impaired glucose metabolism, characterized by increased oxidative stress and immune cell infiltration, also contributes to airway inflammation in asthma. Moreover, the strong link between metabolic dysfunction and cardiovascular diseases, as highlighted by the Global Burden of Disease Study, underscores the necessity of exploring the intersection of asthma and cardiometabolic conditions,^[3]^ given the added cardiovascular burden. The modified cardiometabolic index (MCMI), which integrates waist circumference, height, fasting blood glucose, triglycerides, and high-density lipoprotein cholesterol, provides a more comprehensive assessment of cardiovascular and metabolic risks than traditional measures such as body mass index (BMI) or the cardiometabolic index (CMI).^[4,5]^ Fasting blood glucose, a key component of the MCMI, is closely associated with airway inflammation in asthma, as hyperglycemia induces oxidative stress and immune cell infiltration in the airways. In contrast, BMI only reflects overall adiposity and fails to capture the nuances of metabolic abnormalities associated with asthma. While assessing some cardiometabolic aspects, the CMI omits fasting blood glucose, a critical element in the metabolic-inflammatory cascade of asthma. Thus, MCMI, by including fasting blood glucose along with other cardiometabolic markers, is better positioned to capture asthma-relevant biology. Previous studies have shown that metabolic abnormalities, such as obesity, dyslipidemia, and insulin resistance, are closely associated with the development and progression of asthma.^[6]^ Additionally, newer visceral fat indices, such as CMI and MCMI, are strongly associated with metabolic syndrome.

Notably, while recent studies have found a significant association between CMI and asthma,^[7]^ the exclusion of fasting blood glucose in CMI limits its ability to fully represent the metabolic drivers of asthma. In addition, research on the relationship between MCMI and asthma in adults remains scarce. Therefore, this study aimed to test the association between MCMI (which incorporates fasting blood glucose along with the original components of CMI) and the odds of self-reported asthma in US adults.

## 2. Materials and methods

### 2.1. Data source and study population

#### 2.1.1. Data source

The National Health and Nutrition Examination Survey (NHANES) is a nationally representative survey conducted by the National Center for Health Statistics (NCHS) using stratified multistage probability sampling to assess the health and nutritional status of the US population. NHANES is administered in 2-year cycles; for this study, data from 10 consecutive cycles (1999–2000, 2001–2002, 2003–2004, 2005–2006, 2007–2008, 2009–2010, 2011–2012, 2013–2014, 2015–2016, and 2017–2018) were utilized. The NHANES study protocol was approved by the NCHS Research Ethics Review Board, and the participants provided written informed consent. The use of publicly available deidentified data and the need to obtain consent were waived. This study adhered to the Strengthening the Reporting of Observational Studies in Epidemiology (STROBE) reporting guidelines.

#### 2.1.2. Study population

This cross-sectional study utilized the NHANES data from 1999 to 2018 (https://wwwn.cdc.gov/nchs/nhanes), encompassing the 10 cycles specified above. The NHANES protocols were approved by the NCHS Research Ethics Review Board, and informed consent was obtained from all participants.

**Figure 1.**
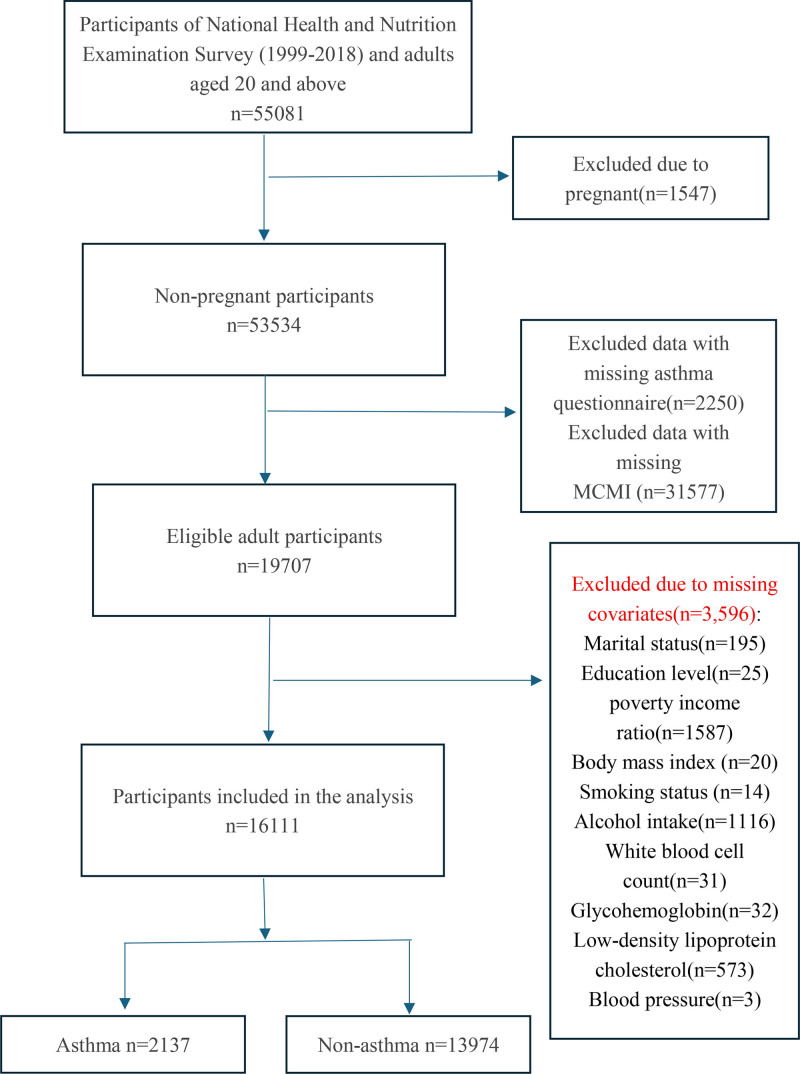
Study’s flow diagram. The item-level missing counts (for marital status, education level, poverty income ratio, etc) are not mutually exclusive. However, in this case, the sum of these individual missing counts equaled the total number of participants excluded because of missing covariates (3596), indicating no overlap among the exclusions for these specific covariates.

Initially, 55,081 individuals were enrolled in the study. To ensure the study’s relevance, we applied exclusion criteria, resulting in the removal of 1547 participants due to pregnancy, 31,577 due to missing MCMI data, and 2250 due to missing asthma-related information. Subsequently, covariate missing values were addressed, and 3596 samples were excluded. Given the large size of the preliminary datasetand the fact that all covariates had <10% missing values, we opted for the direct exclusion of samples with missing covariate data. This process resulted in a final analytical dataset of 16,111 participants. The exclusion procedure is illustrated in Figure 1.

### 2.2. Individuals with asthma

Asthma status was assessed using a questionnaire, in which participants who reported being diagnosed with asthma by a healthcare provider were classified as having asthma.^[8]^ Of the 16,111 participants, 2137 (13.3%) self-reported asthma diagnosis.

### 2.3. Definition and calculation of the MCMI

The modified cardiometabolic index (MCMI) is a composite marker of metabolic dysfunction calculated using the following formula:


$$\begin{array}{l} MCMI=\ln\left(\frac{TG\times FastingBloodGlucose}{HDL\text{\textasciitilde{}}\text{-}C}\right)\times \frac{WC}{Height} \end{array}$$

where TG represents triglycerides, HDL-C is high-density lipoprotein cholesterol, WC is waist circumference, and height is in centimeters.^[5]^ This study examined the relationship between MCMI and the odds of self-reported asthma using NHANES data.

### 2.4. Covariates

The covariates included in the analysis were age, sex, race/ethnicity, marital status, income to poverty ratio (PIR), education level, smoking status, alcohol consumption status, physical activity, body mass index (BMI), hypertension, diabetes mellitus, cardiovascular disease (CVD), chronic bronchitis, emphysema, cancer, HbA1c, white blood cell (WBC) count, and low-density lipoprotein cholesterol (LDL-C).^[9,10]^

Participants were classified according to the following variables:

Race/ethnicity: non-Hispanic White, non-Hispanic Black, Mexican American, other Hispanic, and other races (including multi-racial). Education: less than high school, high school graduate, and above high school graduates. Marital status: married, never married, living with a partner, or other (widowed, divorced, or separated). Income to poverty ratio (PIR): low (≤1.30), medium (1.30–3.50), and high (>3.50). Smoking status was classified as never (<100 cigarettes in a lifetime), former (≥100 cigarettes but quit), or current (≥100 cigarettes and currently smoking).^[11]^ Alcohol consumption was assessed using a 5-level system: never (<12 drinks/lifetime), former (≥12 drinks but none last year), and current (further subdivided into mild, moderate, or heavy based on weekly intake).^[12]^ Physical activity was quantified as total metabolic equivalent (MET)-minutes per week, with zero-activity participants recoded to 0 to minimize missing data.^[13]^ BMI was categorized into 3 groups (<25, 25–30, ≥30), hypertension was defined by elevated blood pressure (≥140/90 mm Hg, excluding zero readings per NHANES protocol), self-reported diagnosis, or antihypertensive medication use. Diabetes was identified by self-reported diagnosis, HbA1c ≥ 6.5%, fasting glucose ≥ 7.0 mmol/L, random glucose ≥ 11.1 mmol/L, or diabetes medication/insulin use.^[14]^ Cardiovascular disease (CVD) was defined as a self-reported history of coronary heart disease, angina, stroke, myocardial infarction, or congestive heart failure.^[15]^ Pulmonary disease included self-reported chronic bronchitis or emphysema, and cancer history was based on self-reported physician diagnosis. All comorbidity data were derived from the NHANES Medical Conditions Questionnaire. Other covariate classification methods will follow the original collection procedures.

### 2.5. Statistical analysis

The distribution of continuous variables was first assessed for normality using Q–Q plots. Variables approximating a normal distribution are presented as mean ± standard deviation, and between-group comparisons for such variables were performed using 1-way ANOVA. Skewed variables are reported as median (interquartile range), with group comparisons performed using the Kruskal–Wallis test. Categorical variables were expressed as frequencies and percentages (%), and comparisons between groups were conducted using the chi-square test. No weighted analysis (including weights, stratification, and PSUs) was performed in this study.

Logistic regression was used to assess the odds ratios (ORs) and 95% confidence intervals (CIs) for the odds of self-reported asthma across the MCMI quartiles (Q1–Q4). Participants were classified into 4 equal groups based on the MCMI: Q1 (0–25th percentile), Q2 (25th–50th percentile), Q3 (50th–75th percentile), and Q4 (75th–100th percentile).

For spline analysis, the model form was rcs (*x*, knots = 4), which indicates using 4 knots for RCS fitting. Knots are the key points of the spline curve that determine the flexibility of the model. These 4 knots are placed at the 5%, 35%, 65%, and 95% percentiles of the data.

Multivariate models were adjusted for potential confounders, including age, sex, and comorbidities of the patients. These confounders were selected based on clinical expertise and findings from the relevant scientific literature.^[16,17]^ Four models were developed for this analysis (Table 2).

**Table 2:** Multivariable logistic regression analyses of modified cardiometabolic index (MCMI) and odds of self-reported asthma in different models.

Variable	Total, n	Event n (%)	Crude model		Model 1		Model 2		Model 3		Model 4	
			OR (95% CI)	*P*-value	OR (95% CI)	*P*-value	OR (95% CI)	*P*-value	OR (95% CI)	*P*-value	OR (95% CI)	*P*-value
MCMI (continuous)	16,111	2137 (13.30)	1.17 (1.11–1.24)	<.001	1.31 (1.24–1.39)	<.001	1.3 (1.23–1.38)	<.001	1.2 (1.12–1.28)	<.001	1.2 (1.12–1.29)	<.001
MCMI (categories)												
Quartile 1	4028	509 (12.60)	1 (Ref)		1 (Ref)		1 (Ref)		1 (Ref)		1 (Ref)	
Quartile 2	4027	486 (12.10)	0.95 (0.83–1.08)	.439	1.12 (0.98–1.28)	.103	1.13 (0.99–1.30)	.072	1.12 (0.97–1.29)	.125	1.12 (0.97–1.29)	.132
Quartile 3	4028	506 (12.60)	0.99 (0.87–1.13)	.920	1.28 (1.12–1.47)	<.001	1.3 (1.13–1.50)	<.001	1.2 (1.04–1.39)	.013	1.20 (1.03–1.39)	.017
Quartile 4	4028	636 (15.80)	1.3 (1.14–1.47)	<.001	1.7 (1.49–1.95)	<.001	1.69 (1.47–1.93)	<.001	1.39 (1.20–1.62)	<.001	1.39 (1.19–1.62)	<.001
*P* for trend				<.001		<.001		<.001		<.001		<.001

Crude model: no other covariates were adjusted.

Model 1: adjusted for age, sex, race.

Model 2: adjusted for age, sex, race, marital status, PIR, education level, smoking status, alcohol intake, physical activity total time.

Model 3: adjusted for age, sex, race, marital status, PIR, education level, smoking status, alcohol intake, physical activity total time, cardiovascular disease, hypertension, diabetes mellitus, chronic bronchitis, emphysema, cancer.

Model 4: adjusted for age, sex, race, marital status, PIR, education level, smoking status, alcohol intake, physical activity total time, cardiovascular disease, hypertension, diabetes mellitus, chronic bronchitis, emphysema, cancer, HbA1c, white blood cell count, and low-density lipoprotein cholesterol.

CI = confidence interval, MCMI = modified cardiometabolic index, OR = odds ratio, PIR = poverty income ratio.

To test the robustness of our results, sensitivity analyses were performed by excluding specific subgroups: participants with chronic bronchitis and current smokers. The stability of our primary findings was confirmed through these analyses, and the corresponding results are presented in Tables 3 and 4.

**Table 3:** Multivariable logistic regression analyses of the association between MCMI and odds of self-reported asthma among individuals without chronic bronchitis.

Variable	Total, n	Event n (%)	Crude model		Model 1		Model 2		Model 3	
			OR (95% CI)	*P*-value	OR (95% CI)	*P*-value	OR (95% CI)	*P*-value	OR (95% CI)	*P*-value
MCMI (continuous)	15,162	1681 (11.10)	1.1 (1.03–1.17)	.004	1.27 (1.19–1.36)	<.001	1.23 (1.14–1.32)	<.001	1.24 (1.15–1.34)	<.001
MCMI (categories)										
Quartile 1	3879	438 (11.30)	1 (Ref)		1 (Ref)		1 (Ref)		1 (Ref)	
Quartile 2	3836	395 (10.30)	0.9 (0.78–1.04)	.159	1.11 (0.96–1.29)	.154	1.11 (0.96–1.29)	.161	1.13 (0.97–1.31)	.126
Quartile 3	3783	387 (10.20)	0.9 (0.77–1.03)	.134	1.24 (1.06–1.44)	.006	1.21 (1.03–1.41)	.018	1.22 (1.04–1.43)	.013
Quartile 4	3664	461 (12.60)	1.13 (0.98–1.30)	.084	1.61 (1.38–1.87)	<.001	1.47 (1.25–1.72)	<.001	1.48 (1.26–1.75)	<.001
*P* for trend				.109		<.001		<.001		<.001

Crude model: No other covariates were adjusted.

Model 1: adjusted for age, sex, race, marital status, PIR, education level, smoking status, alcohol intake, and physical activity total time.

Model 2: adjusted for age, sex, race, marital status, PIR, education level, smoking status, alcohol intake, physical activity total time, cardiovascular disease, hypertension, diabetes mellitus, chronic bronchitis, emphysema, cancer.

Model 3: adjusted for age, sex, race, marital status, PIR, education level, smoking status, alcohol intake, physical activity total time, cardiovascular disease, hypertension, diabetes mellitus, chronic bronchitis, emphysema, cancer, HbA1c, white blood cell count, and low-density lipoprotein cholesterol.

CI = confidence interval, MCMI = modified cardiometabolic index, OR = odds ratio, PIR = poverty income ratio.

**Table 4:** Multivariable logistic regression analyses of the association between MCMI and odds of self-reported asthma among nonsmokers.

Variable	Total, n	Event n (%)	Crude model		Model 1		Model 2		Model 3	
			OR (95% CI)	*P*-value	OR (95% CI)	*P*-value*e*	OR (95% CI)	*P*-value	OR (95% CI)	*P*-value
MCMI (continuous)	8497	1002 (11.80)	1.12 (1.03–1.21)	.006	1.3 (1.2–1.41)	<.001	1.22 (1.11–1.35)	<.001	1.23 (1.11–1.36)	<.001
MCMI (categories)										
Quartile 1	2346	277 (11.80)	1 (Ref)		1 (Ref)		1 (Ref)		1 (Ref)	
Quartile 2	2128	223 (10.50)	0.87 (0.73–1.05)	.159	1.08 (0.89–1.31)	.448	1.03 (0.84–1.25)	.782	1.02 (0.84–1.25)	.832
Quartile 3	2076	231 (11.10)	0.94 (0.78–1.13)	.479	1.3 (1.06–1.58)	.01	1.17 (0.95–1.43)	.139	1.16 (0.94–1.43)	.167
Quartile 4	1947	271 (13.9)	1.21 (1.01–1.45)	.039	1.68 (1.39–2.05)	<.001	1.47 (1.19–1.82)	<.001	1.46 (1.17–1.82)	.001
*P* for trend				.039		<.001		<.001		.001

Crude model: no other covariates were adjusted.

Model 1: adjusted for age, sex, race, marital status, PIR, education level, smoking status, alcohol intake, and physical activity total time.

Model 2: adjusted for age, sex, race, marital status, PIR, education level, smoking status, alcohol intake, physical activity total time, cardiovascular disease, hypertension, diabetes mellitus, chronic bronchitis, emphysema, cancer.

Model 3: adjusted for age, sex, race, marital status, PIR, education level, smoking status, alcohol intake, physical activity total time, cardiovascular disease, hypertension, diabetes mellitus, chronic bronchitis, emphysema, cancer, HbA1c, white blood cell count, and low-density lipoprotein cholesterol.

CI = confidence interval, MCMI = modified cardiometabolic index, OR = odds ratio, PIR = poverty income ratio.

Statistical analyses were carried out using R software (version 4.2.2, http://www.Rproject.org, The R Foundation, Beijing, China) and Free Statistics software version 2.2 (Beijing, China).^[17]^

## 3. Results

### 3.1. Study population and baseline characteristics

Among the 16,111 participants, 2137 (13.3%) reported asthma. The mean age was 50.1 ± 17.9 years, and 50.4% of the patients were male. The racial/ethnic distribution was as follows: 48.4% non-Hispanic White, 19.1% non-Hispanic Black, and 17.3% Mexican American. The median PIR was 2.6 ± 1.6 and the average BMI was 28.8 ± 6.6 kg/m². There were statistically significant differences across the MCMI quartiles (Q1–Q4) in various characteristics. For asthma, the number and percentage of participants with self-reported asthma across the MCMI quartiles were as follows: in Q1, 509 participants (12.6%) reported having asthma; in Q2, 486 participants (12.1%) reported having asthma; in Q3, 506 participants (12.6%) reported having asthma; and in Q4, 636 participants (15.8%) reported having asthma (Table 1).

**Table 1:** Baseline characteristics of study participants according to quartile groups of MCMI.

Characteristic	Total (n = 16,111)	Q1 (n = 4028)	MCMI	Q3 (n = 4028)	Q4 (n = 4028)	*P*-value
			Q2 (n = 4027)			
Sex, n (%)						<.001
Male	8118 (50.4)	1783 (44.3)	2116 (52.5)	2195 (54.5)	2024 (50.2)	
Female	7993 (49.6)	2245 (55.7)	1911 (47.5)	1833 (45.5)	2004 (49.8)	
Age (yr), mean ± SD	50.1 ± 17.9	42.1 ± 17.5	50.3 ± 18.0	53.1 ± 17.4	54.7 ± 16.1	<.001
Race or ethnicity, n (%)						<.001
Non-Hispanic White	7803 (48.4)	2000 (49.7)	1977 (49.1)	1845 (45.8)	1981 (49.2)	
Non-Hispanic Black	3070 (19.1)	950 (23.6)	805 (20)	707 (17.6)	608 (15.1)	
Mexican American	2788 (17.3)	421 (10.5)	611 (15.2)	847 (21.0)	909 (22.6)	
Other Hispanic	1263 (7.8)	240 (6.0)	307 (7.6)	355 (8.8)	361 (9.0)	
Other race (including multi-racial)	1187 (7.4)	417 (10.4)	327 (8.1)	274 (6.8)	169 (4.2)	
Marital status, n (%)						<.001
Married	8709 (54.1)	1869 (46.4)	2208 (54.8)	2370 (58.8)	2262 (56.2)	
Never married	2694 (16.7)	1134 (28.2)	604 (15.0)	504 (12.5)	452 (11.2)	
Living with partner	1196 (7.4)	353 (8.8)	328 (8.1)	256 (6.4)	259 (6.4)	
Other (widowed, divorced, or separated individuals)	3512 (21.8)	672 (16.7)	887 (22.0)	898 (22.3)	1055 (26.2)	
PIR, mean ± SD	2.6 ± 1.6	2.8 ± 1.7	2.7 ± 1.6	2.6 ± 1.6	2.3 ± 1.6	<.001
Education level, n (%)						<.001
<9th grade	4184 (26.0)	722 (17.9)	945 (23.5)	1186 (29.4)	1331 (33.0)	
9–11th grade (including 12th grade with no diploma)	3722 (23.1)	817 (20.3)	931 (23.1)	999 (24.8)	975 (24.2)	
High school graduate or GED: or equivalent	8205 (50.9)	2489 (61.8)	2151 (53.4)	1843 (45.8)	1722 (42.8)	
BMI (kg/m^2^), mean ± SD	28.8 ± 6.6	23.1 ± 3.3	26.8 ± 3.8	29.8 ± 4.5	35.5 ± 6.8	<.001
Smoking status, n (%)						<.001
Never	8497 (52.7)	2346 (58.2)	2128 (52.8)	2076 (51.5)	1947 (48.3)	
Former	4208 (26.1)	739 (18.3)	1055 (26.2)	1140 (28.3)	1274 (31.6)	
Current	3406 (21.1)	943 (23.4)	844 (21.0)	812 (20.2)	807 (20.0)	
Alcohol intake, n (%)						<.001
Never	2184 (13.6)	505 (12.5)	501 (12.4)	541 (13.4)	637 (15.8)	
Former	3032 (18.8)	446 (11.1)	683 (17.0)	870 (21.6)	1033 (25.6)	
Mild	5421 (33.6)	1443 (35.8)	1435 (35.6)	1350 (33.5)	1193 (29.6)	
Moderate	2316 (14.4)	775 (19.2)	584 (14.5)	505 (12.5)	452 (11.2)	
Heavy	3158 (19.6)	859 (21.3)	824 (20.5)	762 (18.9)	713 (17.7)	
PA (MET min/wk), median (IQR)	140.0 (0.0, 561.0)	220.0 (30.0, 761.2)	141.8 (0.0, 572.5)	125.4 (0.0, 510.0)	78.8 (0.0, 420.0)	<.001
CVD, n (%)						<.001
No	14,343 (89.0)	3833 (95.2)	3662 (90.9)	3546 (88.0)	3302 (82.0)	
Yes	1768 (11.0)	195 (4.8)	365 (9.1)	482 (12.0)	726 (18.0)	
Hypertension, n (%)						<.001
No	9283 (57.6)	3153 (78.3)	2481 (61.6)	2100 (52.1)	1549 (38.5)	
Yes	6828 (42.4)	875 (21.7)	1546 (38.4)	1928 (47.9)	2479 (61.5)	
Diabetes mellitus, n (%)						<.001
No	13,198 (81.9)	3880 (96.3)	3642 (90.4)	3278 (81.4)	2398 (59.5)	
Yes	2913 (18.1)	148 (3.7)	385 (9.6)	750 (18.6)	1630 (40.5)	
Chronist bronchitis, n (%)						<.001
No	15,162 (94.1)	3879 (96.3)	3836 (95.3)	3783 (93.9)	3664 (91.0)	
Yes	949 (5.9)	149 (3.7)	191 (4.7)	245 (6.1)	364 (9.0)	
Cancer, n (%)						<.001
No	14,614 (90.7)	3750 (93.1)	3648 (90.6)	3632 (90.2)	3584 (89.0)	
Yes	1497 (9.3)	278 (6.9)	379 (9.4)	396 (9.8)	444 (11.0)	
Pulmonary emphysema, n (%)						<.001
No	15,769 (97.9)	3965 (98.4)	3954 (98.2)	3945 (97.9)	3905 (96.9)	
Yes	342 (2.1)	63 (1.6)	73 (1.8)	83 (2.1)	123 (3.1)	
HbA1c (%), Mean ± SD	5.7 ± 1.0	5.3 ± 0.5	5.5 ± 0.6	5.7 ± 0.9	6.3 ± 1.5	<.001
WBC (×10^9^/L), Mean ± SD	6.8 ± 2.4	6.2 ± 1.9	6.6 ± 2.8	6.9 ± 2.5	7.4 ± 2.1	<.001
LDL-C (mg/dL), mean ± SD	116.1 ± 35.5	104.3 ± 30.9	119.6 ± 34.6	122.5 ± 36.0	118.1 ± 37.2	<.001
Asthma, n (%)						<.001
No	13,974 (86.7)	3519 (87.4)	3541 (87.9)	3522 (87.4)	3392 (84.2)	
Yes	2137 (13.3)	509 (12.6)	486 (12.1)	506 (12.6)	636 (15.8)	

N frequency of participants, mean ± SD for continuous variables, median (Q1–Q4).

BMI = body mass index, CVD = cardiovascular disease, GED = general educational development, HbA1c = glycohemoglobin, IQR = interquartile range, LDL-C = low-density lipoprotein cholesterol, MET = metabolic equivalent, MCMI = modified cardiometabolic index, PA = physical activity, PIR = poverty income ratio, SD = standard deviation, WBC = white blood cell.

### 3.2. Relationship between MCMI and odds of self-reported asthma

In the multivariate logistic regression analysis, the MCMI was analyzed as both a continuous and categorical variable (quartiles, Q1–Q4, with Q1 set as the reference group). After adjusting for confounders, a significant modest positive association was observed between MCMI and the odds of self-reported asthma.

For the continuous MCMI variable, the adjusted ORs for the odds of self-reported asthma gradually changed across different models. Model 1 (adjusted for age, sex, race) revealed that a 1-unit increase in MCMI was significantly associated with a 31.0% higher odds of self-reported asthma, with an OR of 1.31 (95% CI: 1.24–1.39, *P* < .001). Model 2 (additionally adjusted for marital status, poverty income ratio [PIR], education level, smoking status, alcohol intake, and physical activity total time) showed that a 1-unit increase in MCMI was significantly associated with a 30.0% higher odds of self-reported asthma, with an OR of 1.30 (95% CI: 1.23–1.38, *P* < .001). Model 3 (further adjusted for cardiovascular disease, hypertension, diabetes mellitus, chronic bronchitis, emphysema, cancer) demonstrated that a 1-unit increase in MCMI was significantly associated with a 20.0% higher odds of self-reported asthma, with an OR of 1.20 (95% CI: 1.12–1.28, *P* < .001). Model 4 (additionally adjusted for HbA1c, white blood cell [WBC] count, and LDL cholesterol) indicated that a 1-unit increase in MCMI was significantly associated with a 20.0% higher odds of self-reported asthma, with an OR of 1.20 (95% CI: 1.12–1.29, *P* < .001).

**Figure 2.**
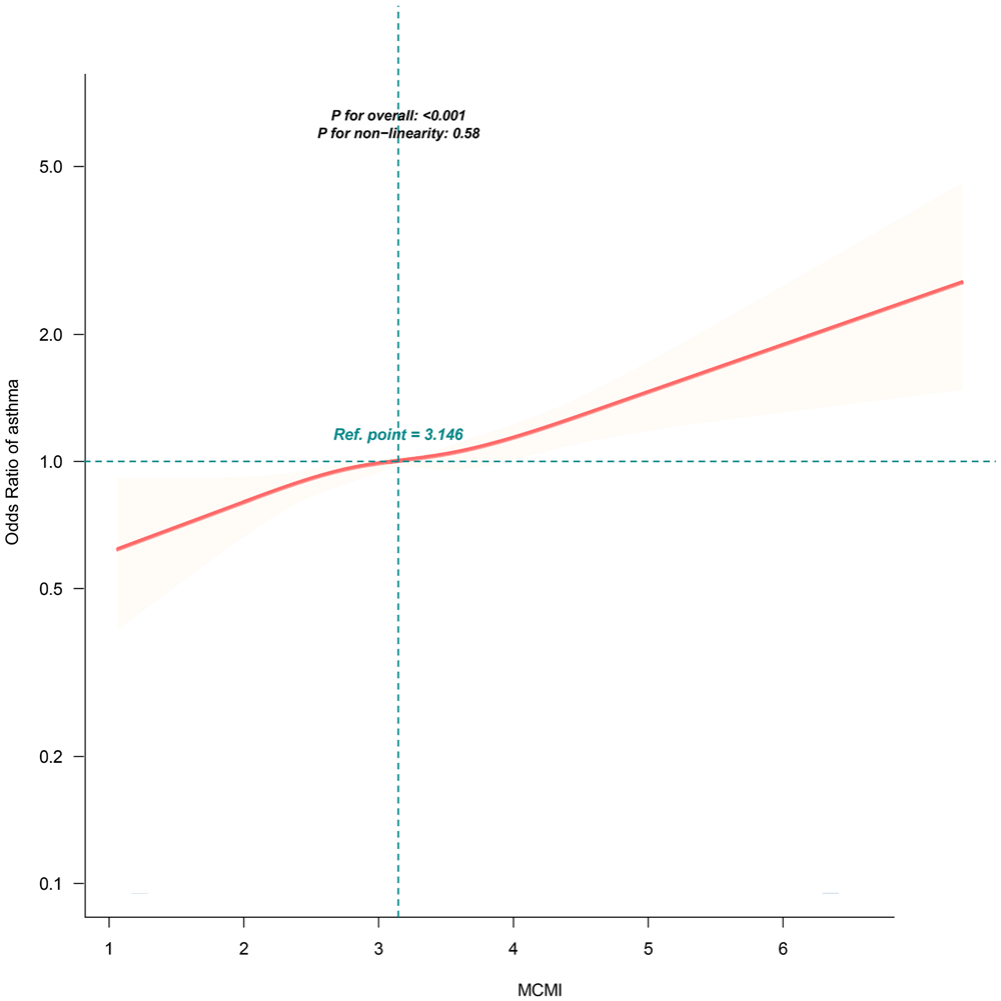
Linear relationship between MCMI and odds of self-reported asthma. The adjustment factors included age, sex, race, marital status, PIR, education level, smoking status, alcohol intake, physical activity total time, cardiovascular disease, hypertension, diabetes mellitus, chronic bronchitis, emphysema, cancer, HbA1c, white blood cell count, and low-density lipoprotein cholesterol. The red and light orange lines represent the estimated values and their corresponding 95% confidence intervals, respectively. The histogram uses its own density scale, independent of the spline axis, to illustrate the distribution of the MCMI values. MCMI = modified cardiometabolic index, PIR = poverty income ratio.

For categorical analysis, the highest MCMI quartile (Q4) had an OR of 1.30 (95% CI: 1.14–1.47, *P* < .001) compared to the lowest quartile (Q1), with a *P* for trend < .001, indicating that higher MCMI levels were associated with increased odds of self-reported asthma (Table 2). Notably, the restricted cubic spline analysis (Fig. 2) revealed a significant linear relationship between the MCMI and the odds of self-reported asthma, with an overall *P* < .001 and *P* for nonlinearity = .58.

**Figure 3.**
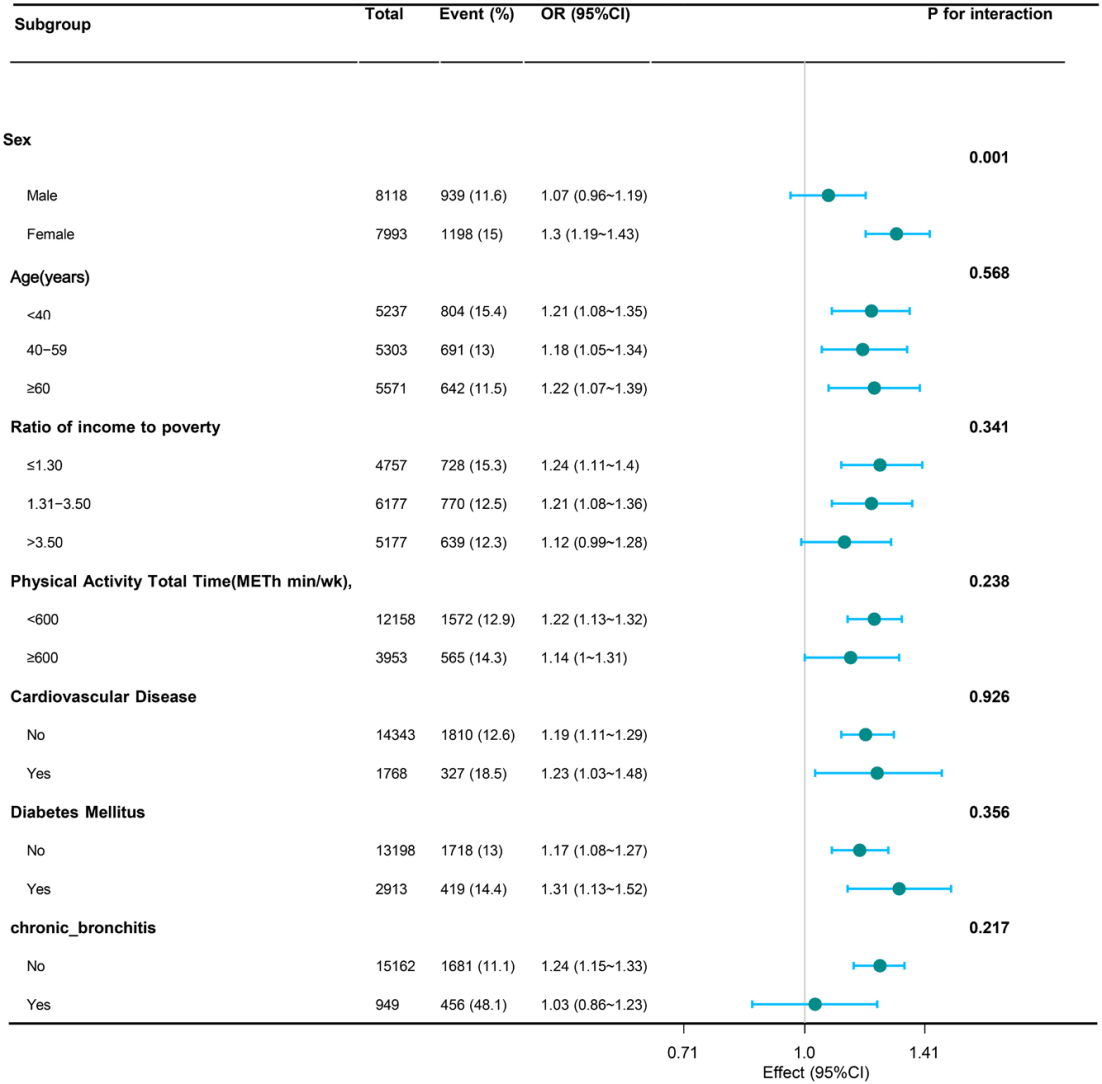
Subgroup analysis of the association between MCMI and the odds of self-reported asthma using multivariable logistic regression. The adjustment factors included age, sex, race, marital status, income to poverty PIR, education level, smoking status, alcohol intake, physical activity total time, cardiovascular disease, hypertension, diabetes mellitus, chronic bronchitis, emphysema, cancer, HbA1c, white blood cell count, and low-density lipoprotein cholesterol. CI = confidence, MCMI = modified cardiometabolic index, OR = odds ratio, PIR = poverty income ratio.

Subgroup analyses were conducted to probe the association between MCMI and the odds of self-reported asthma across diverse demographic and clinical subgroups. We reported the *P*-values for the interaction for all prespecified subgroup factors (Fig. 3). Specifically, the *P*-value for interaction in the “Sex” subgroup was .001, suggesting a statistically significant heterogeneity in the association by sex. For other subgroups (age, income to poverty ratio, total physical activity time, cardiovascular disease, diabetes mellitus, and chronic bronchitis), the *P*-values for interaction were .568, .341, .238, .926, .356, and .217, respectively. These *P*-values were used as tests of heterogeneity to examine whether the association varied across subgroups, rather than as evidence of the reliability of our primary findings.

### 3.3. Sensitivity analysis

Sensitivity analyses confirmed the robustness of the modest positive association between MCMI and the odds of asthma. When excluding participants with chronic bronchitis (n = 15,162, analyzed in Table 3), the association remained stable, with an OR of 1.24 (95% CI: 1.15–1.34, *P* < .001) in model 3 among individuals without chronic bronchitis. Similarly, when excluding smokers (n = 8497, analyzed in Table 4), the association was also stable, with an OR of 1.23 (95% CI: 1.11–1.36, *P* < .001) in model 3 among nonsmokers. These separate sensitivity analyses showed that the relationship remained stable under different analytical conditions.

## 4. Discussion

The modified cardiometabolic index (MCMI) is a comprehensive metric for assessing cardiovascular and metabolic risks. By combining multiple individual indicators, such as triglyceride (TG) levels, fasting blood glucose levels, high-density lipoprotein cholesterol (HDL-C) levels, and waist-to-height ratio, the MCMI provides a more holistic understanding of metabolic health and its association with respiratory conditions, such as asthma. This study aimed to explore the relationship between MCMI and the odds of self-reported asthma in US adults.

Our findings demonstrated a modest positive association between the MCMI and the odds of self-reported asthma, suggesting that metabolic dysfunction, as indicated by the MCMI, is linked to self-reported asthma in the study population. This relationship remained consistent even after adjusting for several confounders, including age, sex, cardiovascular disease, and metabolic indicators such as HbA1c and LDL cholesterol levels. Furthermore, sensitivity analyses that excluded smokers and participants with chronic bronchitis also showed a similar association, further supporting the stability of the link between the MCMI and the odds of self-reported asthma.

These findings are consistent with those of previous studies that have linked metabolic dysfunction, particularly obesity, insulin resistance, and dyslipidemia, to the odds of self-reported asthma.^[7]^ Elevated adiposity, for example, contributes to systemic inflammation, which can exacerbate airway inflammation and lead to airway hyperresponsiveness, a key characteristic of asthma.^[18]^ Obesity-induced inflammation, driven by pro-inflammatory cytokines such as IL-6 and TNF-α, is well documented in the literature and likely plays a role in asthma pathogenesis.^[19,20]^

Metabolic syndrome components, including obesity and dyslipidemia, may promote the occurrence of self-reported asthma by affecting lipid metabolism and activating inflammatory pathways.^[18,21]^

For instance, elevated levels of low-density lipoprotein cholesterol (LDL-C) and triglycerides (TG) can exacerbate airway inflammation by stimulating the production of pro-inflammatory cytokines, which may damage airway epithelial cells and promote asthma symptoms. Dyslipidemia may affect the function of high-density lipoprotein cholesterol (HDL-c), which has anti-inflammatory properties, thereby reducing its protective effect against airway inflammation.^[16]^

Another important observation from this study was the linear relationship between the MCMI and the odds of self-reported asthma. Restricted cubic spline analysis confirmed this linearity, indicating that higher MCMI values corresponded to progressively higher odds of self-reported asthma. This linear pattern supports the potential of the MCMI as a continuous and straightforward indicator for exploring its association with the odds of self-reported asthma in research contexts. While this finding may provide a basis for future inquiries into personalized risk stratification in clinical settings, the cross-sectional design and lack of discrimination or calibration testing mean that definitive conclusions about clinical utility cannot be drawn here.

Our study adds to the growing body of evidence linking metabolic health and respiratory diseases. While previous research has explored the individual components of metabolic syndrome, such as obesity and dyslipidemia,^[22-24]^ the use of the MCMI as a composite measure offers a more comprehensive understanding of the relationship between metabolic dysfunction and asthma. This approach, by integrating multiple risk factors into a single measure, could improve the efficiency of screening and early intervention strategies.

Despite these promising results, this study raises several important questions regarding the underlying mechanisms linking MCMI to asthma. Further research is needed to explore the specific pathways through which MCMI components influence asthma. For example, studies could examine the roles of adipokines, inflammatory markers, and metabolic intermediates in asthma. Additionally, longitudinal studies assessing the MCMI prior to the onset of asthma could provide more conclusive evidence regarding its predictive value.

## 5. Limitation

While this study has several strengths, including the use of a large, nationally representative sample and robust statistical modeling, it also has limitations that should be considered.

First, the cross-sectional design of this study limits our ability to infer causality. Although our findings suggest a modest positive association between MCMI and the odds of self-reported asthma, they do not establish a direct causal relationship. Future studies with longitudinal designs are needed to confirm whether MCMI is associated with changes in the odds of self-reported asthma over time.

Second, reliance on self-reported asthma diagnoses could introduce reporting bias. Although self-reported data are commonly used in large epidemiological studies, clinical diagnoses or objective measures of asthma, such as spirometry, would provide more accurate information. Future studies should incorporate such measures to enhance diagnostic precision.

Finally, although we excluded smokers and individuals with chronic bronchitis from the sensitivity analyses, these groups still represent important subpopulations that could benefit from further research.

## 6. Conclusion

This study provides evidence of a modest positive association between MCMI and the odds of self-reported asthma in US adults. These findings underscore the importance of considering metabolic health in asthma prevention and management. As a composite measure of metabolic dysfunction, the MCMI offers a more comprehensive tool for assessing asthma odds and may be useful in clinical settings for personalized odds stratification.

Although the results are promising, further research is needed to understand the underlying mechanisms linking MCMI to asthma and to assess the potential of MCMI as an early warning indicator for asthma. Longitudinal studies that track MCMI and asthma incidence over time will provide more definitive evidence of their predictive value.

In conclusion, this study contributes to the growing understanding of how metabolic dysfunction, as assessed by the MCMI, may influence asthma odds. By integrating multiple cardiometabolic risk factors, the MCMI has the potential to inform efforts related to asthma; however, further exploration is warranted.

## Acknowledgments

We thank Yuan Fang for conducting the data analyses and writing the manuscript. Man Wang conducted the data analysis and reviewed the manuscript. Yuehu Liu was responsible for data collection. Zi Lin collected the data. Xiaoqin Wang designed the research, conducted the data analysis, and reviewed the manuscript. Qin Zhang designed the research and reviewed the manuscript.

## Author contributions

**Data curation:** Yuan Fang.

**Formal analysis:** Yuan Fang, Yuehu Liu, Zi Lin.

**Methodology:** Yuan Fang, Qin Zhang.

**Project administration:** Qin Zhang.

**Supervision:** Man Wang, Xiaoqin Wang.

**Validation:** Zi Lin, Xiaoqin Wang.

**Writing – original draft:** Yuan Fang.

**Writing – review & editing:** Man Wang, Yuehu Liu, Xiaoqin Wang.
